# Diabetes and hypertension among South Asians in New York and Atlanta leveraging hospital electronic health records

**DOI:** 10.1186/s13098-021-00766-w

**Published:** 2021-12-18

**Authors:** Jeannette M. Beasley, Joyce C. Ho, Sarah Conderino, Lorna E. Thorpe, Megha Shah, Unjali P. Gujral, Jennifer Zanowiak, Nadia Islam

**Affiliations:** 1 Department of Medicine, NYU Grossman School of Medicine, 462 First Avenue CD 673, New York, NY 10016 USA; 2 Department of Mathematics and Computer Science, Emory University, Atlanta, USA; 3 Department of Population Health, NYU Grossman School of Medicine, New York, USA; 4 Department of Family and Preventive Medicine, Emory University School of Medicine, Atlanta, USA; 5 Department of Global Health, Emory University Rollins School of Public Health, Atlanta, USA

**Keywords:** Hypertension, Diabetes, South Asian, Co-morbidity, Electronic health record

## Abstract

**Background:**

Diabetes and hypertension disparities are pronounced among South Asians. There is regional variation in the prevalence of diabetes and hypertension in the US, but it is unknown whether there is variation among South Asians living in the US. The objective of this study was to compare the burden of diabetes and hypertension between South Asian patients receiving care in the health systems of two US cities.

**Methods:**

Cross-sectional analyses were performed using electronic health records (EHR) for 90,137 South Asians receiving care at New York University Langone in New York City (NYC) and 28,868 South Asians receiving care at Emory University (Atlanta). Diabetes was defined as having 2 + encounters with a diagnosis of diabetes, having a diabetes medication prescribed (excluding Acarbose/Metformin), or having 2 + abnormal A1C levels (≥ 6.5%) and 1 + encounter with a diagnosis of diabetes. Hypertension was defined as having 3 + BP readings of systolic BP ≥ 130 mmHg or diastolic BP ≥ 80 mmHg, 2 + encounters with a diagnosis of hypertension, or having an anti-hypertensive medication prescribed.

**Results:**

Among South Asian patients at these two large, private health systems, age-adjusted diabetes burden was 10.7% in NYC compared to 6.7% in Atlanta. Age-adjusted hypertension burden was 20.9% in NYC compared to 24.7% in Atlanta. In Atlanta, 75.6% of those with diabetes had comorbid hypertension compared to 46.2% in NYC.

**Conclusions:**

These findings suggest differences by region and sex in diabetes and hypertension risk. Additionally, these results call for better characterization of race/ethnicity in EHRs to identify ethnic subgroup variation, as well as intervention studies to reduce lifestyle exposures that underlie the elevated risk for type 2 diabetes and hypertension development in South Asians.

**Supplementary Information:**

The online version contains supplementary material available at 10.1186/s13098-021-00766-w.

## Background

Over two-thirds of United States (US) adults with diabetes have hypertension, and half are not meeting blood pressure (BP) goals despite having antihypertensive treatment [1]. Hypertension is associated with a two times greater risk for cardiovascular disease events and mortality among adults with diabetes [2]. Diabetes disparities are pronounced among South Asians compared to other race/ethnic groups in the United States [3]. National studies have found US South Asians have the highest age-adjusted burden of diabetes (23%) compared to whites (6%), Chinese Americans (13%), Latinos (17%), and African Americans (18%) [4]. Further, among South Asians with diabetes, 72% also have co-morbid hypertension [5].

The purpose of this cross-sectional analysis is to compare diabetes and hypertension burden among South Asians receiving care at two large academic medical centers in New York City (NYC) and Atlanta. Understanding disparities across these populations may help disentangle issues related to environmental factors, health services, and/or immigration by region. These data will provide the foundation for discussion regarding similarities and differences between the populations across sites to inform the development of intervention strategies to reduce the burden of diabetes complications among South Asians in the US. These findings will facilitate bidirectional future research efforts to identify and address disparities across the populations. Given the rapid growth of South Asians in these regions and their high burden of diabetes and hypertension, a critical need exists to tailor, translate, and disseminate evidence-based interventions to maximize impact in ameliorating co-morbid cardiovascular disease disparities [6].

## Methods

### Study population

We utilized electronic health record (EHR) data on the Epic platform at New York University (NYU) Langone Health and the Cerner platform at Emory University to measure patient burden of chronic diseases. Eligibility criteria of patients at each of the sites were: (1) South Asian ethnicity (identified by common South Asian surnames, race/ethnicity as listed in the EHR, or language preference); (2) 18 + years of age or older; and (3) was seen by a primary care physician at NYU Langone between 2014 and 2019 or Emory between 2014 and 2017. Individuals were excluded if they were pregnant at the time of visit. This study was approved by the Institutional Review Board at NYU Grossman School of Medicine.

### Metric definitions

#### Identifying South Asian patients

We derived a list of 12,907 South Asian surnames by compiling a list of names from Social Security Administration data [7], death certificate data [8], and prior studies conducted among South Asians [9, 10]. For race/ethnicity as listed in the EHR, selection criteria included Asian Indian, Bangladeshi, Pakistani, or Sri Lankan. For preferred language, selection criteria included Bengali, Gujarati, Hindi, Kannada, Kashmiri, Malayalam, Nepali, Pakistani, Punjabi, Sindhi, Sinhalese, Tamil, or Urdu.

Rule-based algorithms developed for EHR were used to define diabetes and hypertension, as fully described elsewhere [11, 12]. Briefly, burden estimates were defined by combining: ICD-9-CM and ICD-10-CM diagnostic codes, lab results or vitals, or relevant medications (Additional file 1: Table S1). Biologically implausible measurements for vitals and labs, such as systolic BP outside the range of 140–250 mmHg or diastolic BP outside the range of 90–150 mmHg, were excluded from these algorithms.

### Sensitivity analyses

Among NYC data, sensitivity analyses were conducted to restrict the sample to those identified as Asian Indian, Bangladeshi, Pakistani, or Sri Lankan race/ethnicity using the standard fields in the EHR. Modifications tested were based on variations in definitions seen in the literature and hypothesized concerns with data quality.

### Statistical analyses

Proportion of individuals with diabetes, hypertension, and comorbid diabetes and hypertension were estimated by site and sex and were age-adjusted using direct standardization with the 2000 census as the standard population [13]. Participant characteristics were stratified by sex and compared by site using likelihood ratio χ^2^ tests. Analyses were performed using SAS, version 9.4 (SAS Institute, Cary, NC).

## Results

The sample included 90,137 individuals receiving care at NYU Langone in NYC, and 28,868 individuals receiving care at Emory University in Atlanta (Table 1). Mean age was 48.8 (SD 17.4) years among women and 50.1 (SD 17.5) years among men in NYC compared to a mean age of 55.6 (SD 19.4) years among Atlanta women and 58.4 (SD 19.5) years among Atlanta men. Two-thirds of each sample had private insurance.

Using surnames as the criteria to identify South Asians, the most commonly reported first race/ethnicity in the NYC sample was Asian Indian (25.5% among women and 27.7% among men). Using the same list of surnames to select the Atlanta sample, Asian was the fourth most commonly reported first race/ethnicity (20.3% among women and 22.9% among men) after Caucasian, African American, and Unknown.

**Fig. 1:**
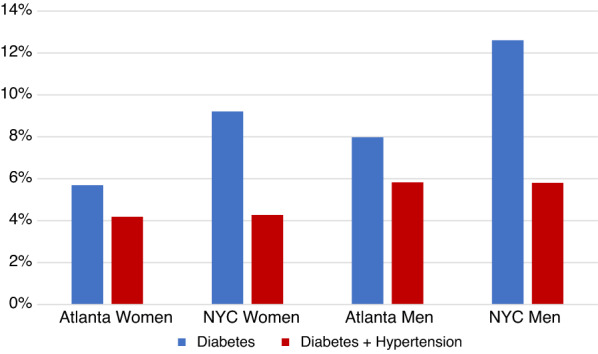
Age-adjusted proportion with diabetes and co-morbid hypertension by sex and region

After age-adjustment, one-fifth (19.4% of women and 23.1% of men) of NYC patients had a hypertension diagnosis, while approximately a quarter of Atlanta patients had a hypertension diagnosis (23.3% of women and 26.5% of men) (Table 2). Age-adjusted diabetes burden was 9.2% among women and 12.6% among men in NYC, compared to 5.7% among women and 8.0% among men in Atlanta (Table 3). P-values for all statistical comparisons by location and sex were significant (p < 0.001). In Atlanta, 75.6% of those with diabetes had comorbid hypertension compared to 46.2% in NYC. Figure 1 underscores the large proportion of those with diabetes and co-morbid hypertension.

**Table 1:** South Asian patient population by study site, 2014–2019*

Characteristic	Women		Men	
	Atlanta (n = 14,916)	New York City (n = 50,815)	Atlanta (n = 11,825)	New York City (n = 39,322)
South Asian inclusion criteria, n (%)				
South Asian surname	14,916 (100)	43,520 (85.6)	11,825 (100)	34,085 (86.7)
South Asian race/ethnicity		15,730 (31.0)		13,393 (34.1)
South Asian language		2408 (4.7)		1424 (3.6)
South Asian surname and race/ethnicity		8995 (17.7)		8452 (21.5)
South Asian surname and language		1952 (3.8)		1191 (3.0)
South Asian surname, language, and race/ethnicity		1200 (2.4)		749 (1.9)
		1000 (2.0)		647 (1.6)
Age, mean (SD)	56.9 (18.3)	48.8 (17.4)	60.2 (17.5)	49.0 (17.0)
Race/ethnicity, n (%)				
American Indian or Alaska Native	298 (2.0)	317 (0.6)	271 (2.3)	228 (0.6)
Asian	3088 (20.7)	18,607 (36.5)	2786 (23.6)	15,375 (39.1)
Black or African American	3488 (23.4)	4187 (8.2)	1872 (15.8)	2509 (6.4)
Hispanic or Latino	4 (0.03)	n/a	3 (0.03)	n/a
Native Hawaiian or Other Pacific Islander	129 (0.9)	296 (0.6)	82 (0.7)	171 (0.4)
White	4661 (31.2)	9268 (18.2)	3526 (29.8)	6472 (16.5)
Other	141 (0.9)	9298 (18.3)	135 (1.1)	7766 (19.8)
Unknown	3107 (20.8)	8813 (17.4)	3150 (26.6)	6761 (17.2)
Language spoken				
Bengali	102 (0.7)	1005 (2.0)	53 (0.4)	728 (1.9)
English	11,400 (76.4)	44,658 (87.9)	8764 (74.1)	35,404 (90.0)
Gujarati	89 (0.6)	80 (0.2)	56 (0.5)	37 (0.09)
Hindi	155 (1.0)	443 (0.9)	109 (0.9)	242 (0.6)
Kannada	0 (0.0)	1 (0.01)	0 (0.0)	0 (0.0)
Malayalam	6 (0.04)	35 (0.07)	4 (0.03)	17 (0.04)
Nepali	65 (0.4)	89 (0.18)	51 (0.4)	73 (0.2)
Punjabi	13 (0.09)	144 (0.28)	0 (0.0)	95 (0.24)
Tamil	n/a	17 (0.03)	n/a	9 (0.02)
Urdu	68 (0.5)	772 (1.5)	32 (0.3)	333 (0.9)
Other	449 (3.0)	3569 (7.0)	1122 (9.5)	2382 (6.0)
Not recorded	2569 (17.2)	0 (0.0)	2458 (20.8)	0 (0.0)
Insurance status				
Medicare	3326 (22.3)	6048 (11.9)	2742 (23.2)	5017 (12.8)
Medicaid	932 (6.2)	9817 (19.3)	502 (4.2)	6783 (17.3)
Private Insurance (HMO, PPO, POS, Indemnity, EPO, Managed Care)	9915 (66.5)	33,159 (65.3)	7838 (66.3)	25,556 (65.0)
Other (self pay, workers comp, no fault, child health plus)	743 (5.0)	465 (0.9)	743 (6.3)	760 (1.9)
Missing	n/a	1326 (2.6)	n/a	1206 (3.1)
Smoke cigarettes, n (%)				
Yes	765 (5.1)	3470 (6.8)	1113 (9.4)	6546 (16.7)
No	11,274 (75.6)	46,768 (92.0)	8597 (57.6)	32,277 (82.1)
Missing/NA	2877 (19.3)	577 (1.1)	2115 (14.2)	499 (1.3)

*All variables were collected using electronic health record

**Table 2:** Outcome* comparisons by study site

Outcome	Atlanta, n (%**)	New York City, n (%)**	p-value***
Diabetes	2447 (6.7)	11,542 (10.7)	< 0.0001
Hypertension	8233 (24.7)	20,773 (20.9)	< 0.0001
Proportion of those with diabetes having comorbid hypertension	1849 (75.6)	5335 (46.2)	< 0.0001

*EHR definitions described in Additional file 1: Table S1

**Age adjusted burden using the 2000 Census as the reference population

***p-values ≤ 0.0001 using chi-squared tests

**Table 3:** Outcome* comparisons by sex and study site

Outcome	Women		Men		p-value for women***	p-value for men***
	Atlanta (n = 14,916)	New York City (n = 50,815)	Atlanta (n = 11,825)	New York City (n = 39,322)		
	n (%)**	n (%)**	n(%)**	n(%)**		
Diabetes	1119 (5.7)	5383 (9.2)	1328 (8.0)	6158 (12.6)	< 0.0001	< 0.0001
Hypertension	4285 (23.3)	11,232 (19.4)	3948 (26.5)	10,036 (23.1)	< 0.0001	< 0.0001
Proportion of those with diabetes having comorbid hypertension	852 (76.1)	2510 (46.6)	997 (75.1)	2825 (45.9)	< 0.0001	< 0.0001

*EHR definitions described in Additional file 1: Table S1

**Age adjusted burden using the 2000 Census as the reference population

***p-values ≤ 0.0001 using chi-squared tests

## Discussion

Diabetes burden in the US South Asian community is high, with national and regional data revealing the highest burden of diagnosed diabetes among Asian Indians compared to other Asian groups [14] and compared to non-Hispanic whites [15–19],^20^, ^21^. Research also indicates that South Asians living in the US have a higher burden of hypertension compared to some other race/ethnic populations [22, 23]. Our study found significant differences in the burden of diagnosed diabetes and hypertension in South Asians receiving care in NYC compared to Atlanta. Specifically, there was a higher burden of diagnosed diabetes in NYC compared to Atlanta (10.7% compared to 6.7% respectively). The burden of diabetes amongst South Asians in NYC and Atlanta reported in our study is lower than that reported in previous research. Prior community health surveys (NYC Community Health Resources and Needs Assessment 2013–2016 and NYC Community Health Survey 2013–2017) demonstrated that Asian Indians in NYC have a tremendous burden of self-reported diabetes (21%) [3]. Additionally, the burden of self-reported diabetes diagnoses among South Asians of normal weight (using adjusted-BMI guidelines for Asians) in NYC is more than triple the rates of diabetes among non-Hispanic whites of normal weight (10.2% vs 2.9%, respectively) [24]. Furthermore, in a community-based survey of Asian Indians in Atlanta, the self-reported burden of diabetes was 18.3%, nearly four times as high as non-Hispanic whites and twice as high as Hispanics [25]. Among those who reported diabetes, there was a > 3.5 odds of having co-morbid hypertension. This population further had higher burden of stroke (2.77%) compared to whites (2.12%) [26].

Similarly, we found differences in the burden of hypertension between South Asians receiving health care in NYC compared to Atlanta, with those in Atlanta having a higher burden of 24.7% compared to 20.9% in NYC. The burden of hypertension reported in our study is lower than that found in a representative survey of both diagnosed and undiagnosed hypertension in NYC, which found a burden of hypertension in South Asians of 43% [23].

Nationwide, 21.4% of people with diabetes are undiagnosed [27]. Differential access to care may partially explain the higher burden of diabetes in NYC versus Atlanta, as New York has expanded their Medicaid program to cover all people with household incomes below 133% of the federal poverty level, while Georgia has not. The lower burden of diabetes and hypertension amongst South Asians in our study compared to previous findings could be due to the fact that our data was collected from two large, private hospital systems, which provide patients with more consistent access to healthcare and may therefore make it more likely for patients to receive preventative care. It is interesting to note that there were significant differences in the burden of diabetes and hypertension between South Asians in the two regions of the US and additional research is necessary to assess differences in regional risk factors that could contribute to this disparity. In addition, a considerable number of South Asians in our study (n = 7184) had a diagnosis of comorbid diabetes and hypertension. Thus, scalable and translatable interventions that promote diabetes management and hypertension control in this population may have significant potential for public health impact and reducing disparities across the US and South Asia.

We acknowledge several limitations. One limitation of this analysis to consider is misclassification error of South Asian origin based on surnames. Prior work has suggested positive predictive values of surname lists ranging from 74 to 91% for Indian surnames in the US [7] and 89.3% among South Asians in Canada [8]. We conducted sensitivity analyses limiting the sample to those reporting South Asian race/ethnicity in the NYC sample, and diabetes burden was 11.2%, suggesting risk may be underestimated in these analyses. The cross-sectional nature of these analyses preclude us from examining change over time, but they provide an initial snapshot of regional differences in diabetes burden by region from the perspective of two academic health centers. Selection bias is another key limitation of our analyses. Prior work has demonstrated the study sample is more economically privileged compared to NYC as a whole due to the nature of the NYU patient population [11], and the patient population at Emory is also a selected sample. Several other limitations of using EHR data in clinical research have been previously noted [28–30], such as differences in procedures for documenting care across systems that could contribute to systematic differences in disease estimates across sites. Finally, we were unable to obtain data on potentially informative covariates such as obesity (BMI was frequently missing in the Atlanta data), diet, physical activity, immigration history, and socioeconomic status, thus limiting the range of analyses we were able to conduct.

Asian Americans currently compose 5% of the US population and approximately 32% of the immigrants entering the country [31]. The US Census Bureau projects that by 2060, the number of Asian Americans nationally will grow to over 39 million, approximately 9.3% of the US population [32, 33]. In NYC, the South Asian community grew by 49% from 2000 to 2010 (216,179 to 323,675, respectively). South Asians also make up the largest Asian American subgroup in the Atlanta metro area. Across South Asian groups, a significant portion of the community live in poverty (ranging from 17% of Asian Indians to 32% of Bangladeshis), have limited English proficiency impacting access to care (ranging from 25% of Asian Indians to 53% of Bangladeshis), and have poor access to culturally appropriate community resources [34–37].

## Conclusions

This work highlights the need for health systems to collect more accurate demographic data on patients they care for to improve our population health. Currently, disaggregated data by race/ethnicity on disease prevalence are not available for all states; for example, diabetes prevalence among Asians is unavailable for Georgia [38]. Even so, we found evidence that two key chronic conditions—hypertension and diabetes—represents a significant burden of disease among South Asians in NYC and Atlanta. There is also a need for interventions tailored for South Asian subgroups that can reduce the burden of hypertension and diabetes. For example, a community health worker intervention that was successful in NYC is currently being tested among South Asians in Atlanta [39], and further dissemination of the intervention could reduce diabetes and hypertension burden among South Asians in the US.

## Supplementary Information

**Additional file 1: Table S1.** Outcome definitions and list of ICD-10 codes and medications.

## Data Availability

The datasets used and/or analyzed during the current study are available from the corresponding author on reasonable request.

## Abbreviations

BP: Blood pressure
EHR: Electronic health record
NYC: New York City
NYU: New York University
SD: Standard deviation
US: United States
