# Supplementary material for: Diabetes and hypertension among South Asians in New York and Atlanta leveraging hospital electronic health records
Source: Diabetol Metab Syndr. 2021 Dec 18;13:146. doi: 10.1186/s13098-021-00766-w (PMC8684279; doi:10.1186/s13098-021-00766-w)
Supplement: Supplementary file 1 — Additional file 1: Table S1. Outcome definitions and list of ICD-10 codes and medications. [file 13098_2021_766_MOESM1_ESM.docx]

Table S1. Outcome definitions and list of ICD-10 codes and medications

Diabetes: Having 2+ encounters with a diagnosis of diabetes† *or*

have a diabetes medication prescribed* (excluding Acarbose/Metformin) *or*

having 2+ abnormal A1C levels (≥6.5% and ≤30%)** and 1+ encounters with a diagnosis of diabetes***

Hypertension: Having 3+ BP readings of systolic BP ≥130 mmHg*** or diastolic BP ≥80 mmHg*** *or*

2+ encounters with a diagnosis of hypertension† *or* had anti-hypertensive medication prescribed*

*In past calendar year.

**In past two calendar years.

***Ever in medical record.

| **Condition** | **ICD-10 Diagnostic codes** | **Medications** | **Medication names** | **LOINC Codes** |
| --- | --- | --- | --- | --- |
| Hypertension | I10, I11.0, I11.9, I12.9, I13.0, I13.10, I67.4, H35.031-H35.033, H35.039 | Therapeutic or pharmaceutical class/subclass of thiazide, calcium channel, beta-blocker, ACE I, or angiotensin | <https://www.heart.org/en/health-topics/high-blood-pressure/changes-you-can-make-to-manage-high-blood-pressure/types-of-blood-pressure-medications> | Not Applicable |
| Type 2 Diabetes Mellitus | E11.00, E11.01, E11.21, E11.22, E11.29, E11.311, E11.319, E11.321, E11.329, E11.331, E11.339, E11.341, E11.349, E11.351, E11.359, E11.36, E11.39, E11.40-E11.44, E11.49, E11.51, E11.52, E11.59, E11.610, E11.618, E11.620-E11.622, E11.628, E11.630, E11.638, E11.641, E11.649, E11.65, E11.69, E11.8, E11.9, E13.00, E13.01, E13.10, E13.11, E13.21, E13.22, E13.29, E13.311, E13.319, E13.321, E13.329, E13.331, E13.339, E13.341, E13.349, E13.351, E13.359, E13.36, E13.39, E13.40-E13.44, E13.49, E13.51, E13.52, E13.59, E13.610, E13.618, E13.620, E13.621, E13.622, E13.628, E13.630, E13.638, E13.641, E13.649, E13.65, E13.69, E13.8, E13.9 | Therapeutic class of Antihyperglycemic, excluding medication names of Metformin and Acarbose | <https://dtc.ucsf.edu/types-of-diabetes/type2/treatment-of-type-2-diabetes/medications-and-therapies/type-2-non-insulin-therapies/table-of-medications/> | **A1C:** 4548-4, 7426-0, 4549-2, 17856-6, 59261-8, 71875-9, 62388-4, 4637-5, 55454-3, 41995-2, 17855-8 |
